# Questionnaire study on the utility of autopsy case conferences related to emergency medicine practices

**DOI:** 10.1097/MD.0000000000015315

**Published:** 2019-04-26

**Authors:** Hideyuki Maeda, Takako Tsujimura, Ken-ichi Yoshida

**Affiliations:** a Department of Forensic Medicine, Tokyo Medical University, Shijuku, Shinjuku-ku, Tokyo; b Department of Japanese Linguistics, School of Medicine, Tokyo Women's Medical University, Kawatacho, Shinjyuku-ku, Tokyo, Japan.

**Keywords:** autopsy information, conference, discrepancy of diagnosis, emergency medicine, imaging diagnosis

## Abstract

We examined whether and how conferences on cases of medico-legal autopsy after emergency medicine (EM) practices improved the diagnostic accuracy and expertise of emergency medicine practitioners (EMPs) and forensic pathologists (FPs); we also examined the necessity of imaging in autopsy diagnoses. We additionally discuss whether imaging could replace autopsy.

An unsigned, self-administered questionnaire was distributed to the attendees of monthly case conferences during which EMPs and FPs discussed EM-associated autopsy cases. The questionnaire addressed the following 6 questions: was the conference useful for forensic medicine or EM practices, was autopsy necessary for each case, were the autopsy and clinical diagnoses consistent, was imaging necessary for autopsy diagnosis, and should autopsy results be disclosed to the public. Participants were autopsy operators, third-party EMPs, and FPs, primarily from universities in and near Tokyo.

Fifty-two cases were discussed; more than 80% of the attendees acknowledged the usefulness of autopsy and the conferences, and 33.6% corrected their diagnoses by considering autopsy information. Major clinical misdiagnoses were corrected by autopsy in 35.3% of cases, including procedure-related hemorrhage, intoxication, asphyxia, fat embolism, diabetes, organ injuries, and subarachnoid hemorrhage (SAH). Approximately 75% of the attendees recognized the usefulness of imaging for autopsy. However, in a series of four SAH cases, the clinical diagnoses were corrected after the conferences more often by EMPs than by FPs. In a violence-related case, false legal judgment was prevented because the conference discussion corrected the clinical diagnosis from traumatic to natural.

In conclusion, the conference improved the accuracy and expertise of diagnoses provided by EMPs and FPs; conference participation led to the correction of major clinical misdiagnoses as well as that of the first diagnoses issued by attendees in more than one-third cases. The usefulness of imaging for autopsy was acknowledged by two thirds of the attendees. Our results also suggested that imaging cannot replace autopsy in deaths related to procedure or violence and in several categories of deaths such as intoxication and asphyxia.

## 1 Introduction

Emergency medicine practitioners (EMPs) routinely treat undiagnosed cases without the availability of adequate information; these include cases of various injuries and intensive care cases with complex clinical backgrounds. Medico-legal autopsies are performed in cases where death is due to unnatural, un-witnessed, unexpected, or medical practice-associated causes. A series of studies have documented substantial discrepancies between clinical (imaging) diagnoses and autopsy findings.^[1,2]^ Autopsies are required for the appraisal and improvement of emergency medicine (EM) practices, because major diagnostic errors have been identified in 31.7% of cases involving intensive care unit (ICU) patients.^[3]^ The decline in histopathological autopsy rates worldwide and the extremely low medico-legal autopsy rate in Japan (1.6% of overall deaths in 2012) have promoted the idea that radiological imaging-based diagnoses can replace autopsies. This idea was generally viewed positively by EMPs but negatively by forensic pathologists (FPs).^[4]^

In Japan, the law (Code of Criminal Procedure) prohibits the disclosure of information related to medico-legal autopsies before disclosure in a criminal court. Recently, our survey of EMPs showed that many respondents complained of a lack of feedback regarding the medico-legal autopsy results of cases, even though EMPs deemed such information to be useful for the assessment of their practices and for providing explanations to bereaved families.^[5]^ Additionally, a concern was raised about the possibility of incorrect single-expert opinions by EMPs or FPs and of prosecutors’ misunderstanding of these opinions, which can lead to inappropriate criminal prosecution.

Our departments hold monthly case conferences after arguments with the District Prosecution Department. In these conferences, EMPs and FPs discuss medico-legal autopsy cases related to EM practices and some medical practice-associated deaths (MPADs). We performed a questionnaire study to clarify the necessity of autopsies and conference discussions, discrepancies between clinical and autopsy diagnoses, and substitutability of imaging for autopsy after discussing each case. We also examined the role of conference discussions in the prevention of similar misdiagnoses and inappropriate legal judgments.

## 2 Methods

### 2.1 Conferences and subjects

We held 17 monthly case conferences between May 2012 and December 2013, in which 52 medico-legal autopsy cases consequent to EM practices or MPADs were discussed. Most of the attendees were EMPs and FPs who were employed at universities in and around the Tokyo metropolitan area (because of the convenience of regular attendance at the conference). Additionally, we obtained permission for members of the Tokyo District Prosecution Department to attend the conferences.

In discussions regarding a few MPADs, 1–2 experts appraised the clinical courses, and several experts attended the conferences. After the discussion of each case, an unsigned, self-administered questionnaire was distributed to and collected from the attendees. This study was approved by the Ethics Committee of the Graduate School of Medicine, The University of Tokyo, with the understanding that personal and institutional privacy was strictly protected. Given the legal limitations in Japan, we could not obtain informed consent from the bereaved families.^[4]^

Accordingly, we requested police officers to provide the bereaved families with leaflets that explained organ retention, potential uses for research, and right to refuse research use.

### 2.2 Questionnaire

The questionnaire included the questions listed in Table 1, and the answers to each question included 2 positive and 2 negative choices. Each attendee provided “clinical diagnoses” based on the case history and imaging study results, but without access to autopsy information. “Autopsy diagnoses” were provided by the attendees after conference discussions with autopsy findings in addition to analysis of the clinical course. For each case, attendees (EMPs, FPs) were questioned on the usefulness of the case conference, necessity of autopsy, comparison of clinical and autopsy diagnoses, necessity of imaging for autopsy diagnosis, and necessity of disclosure of autopsy information. The percentage of “yes” and “no” responses provided by EMPs and FPs was calculated. For each case, the percentage of EMPs/FPs who changed their diagnosis after autopsy information was disclosed, was calculated to determine cases in which autopsy was considered as a requirement. We then analyzed the reasons for the correction of diagnoses by autopsy, by referring to medical records (e.g., free descriptions) and recalling the discussions that took place at the conferences. We excluded answer sheets with responses to less than 80% of all questions. Of the 1446 answer sheets collected in total, 1044 were analyzed.

**Table 1:**
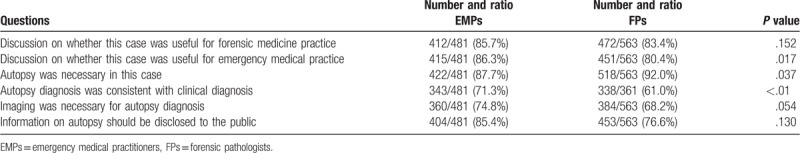
Aggregate answers to questions pertaining to all eligible cases on the necessity of discussion, autopsy, imaging, and information disclosure.

### 2.3 Statistical analysis

Differences between responses provided by EMPs and FPs were analyzed using chi-square test or Fisher's exact test, and the significance level was set at *P* < .05. Statistical analyses of the completed responses were performed using Statistical Package for the Social Sciences (SPSS) version 21 (SPSS Inc., Chicago, IL).

## 3 Results

**Figure 1:**
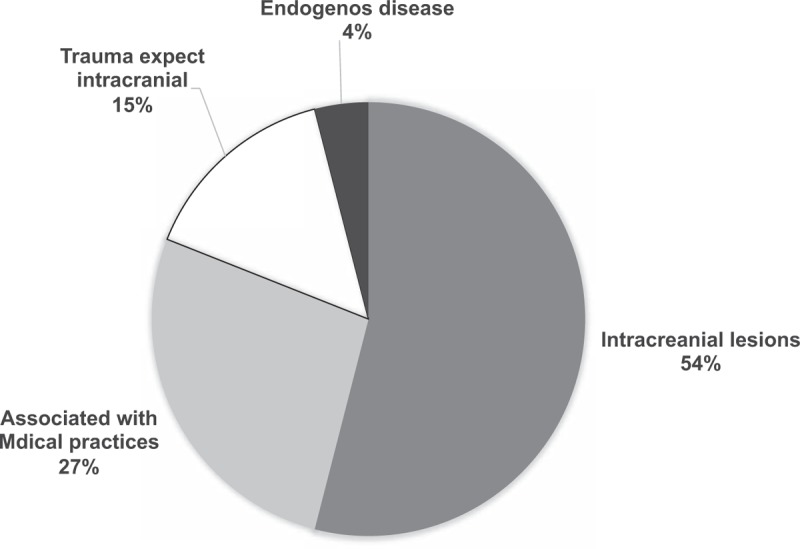
Cases in which imaging was considered to be required for autopsy diagnosis.

EMPs and FPs comprised 38% and 33% of the overall respondents, respectively, and each conference had 30 ± 2.1 attendees. Table 1 shows that the proportion of EMPs who acknowledged that the usefulness of conference discussions with regard to EM practices was slightly higher than that of FPs (86.3% vs. 80.4%; *P* = .017), whereas similar proportions of EMPs and FPs noted the usefulness of the conferences with regard to FP practices (85.7% vs. 83.4%). In addition, the necessity of autopsy was noted by approximately 90% of the attendees (92.0% vs. 87.7%; *P* = .037). The disagreement between the groups in terms of autopsy necessity was especially high with respect to a subarachnoid hemorrhage (SAH) case (FPs, 100% vs. EMPs, 50%; *P* = .018; case 1 in Table 2). Overall, concordance rates of the clinical and autopsy diagnoses were approximately 70% among the EMPs (71.3%) and FPs (68.2%). Meanwhile, large proportions of both the EMPs (85.4%) and FPs (76.6%) noted the necessity of disclosing autopsy information to the public. Approximately 70% of EMPs and FPs (74.8% vs. 68.2%, respectively; *P* = .054) acknowledged the usefulness of imaging for autopsy diagnosis. Among the participants with ratios of positive answers above the median value of 83%, the attendees thought that imaging was required for autopsy diagnoses of intracranial lesions (54%) and MPADs (27%) (Fig. 1). Given that intracranial lesions can be detected by imaging, it is notable that many respondents agreed with the requirement of imaging for autopsy diagnoses, and the reasons for this will be discussed below with respect to SAH cases.

**Table 2:**
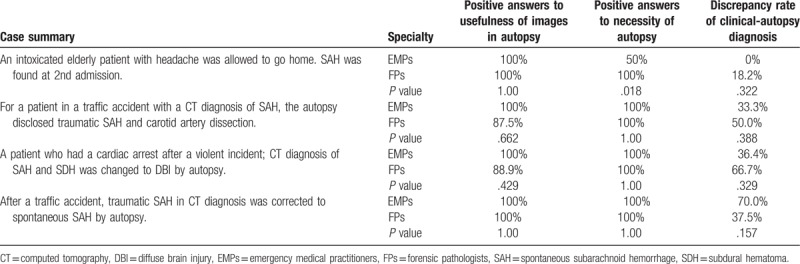
Acknowledgement of usefulness of images for autopsy, necessity of autopsy, and clinical-autopsy discrepancy rate in subarachnoid hemorrhage cases.

Because the high discrepancy rate of clinical-autopsy diagnoses in certain cases underscores the autopsy requirement for such cases, we analyzed 15 cases with a >50% overall clinical-autopsy diagnostic discrepancy rate (Table 3). In this study, “clinical diagnosis” and “autopsy diagnosis” were provided by each EMP or FP without and with autopsy information, respectively, after the conference discussion. Accordingly, the discrepancy rate reflects correction of the diagnosis discussion of autopsy findings for each attendee.

**Table 3:**
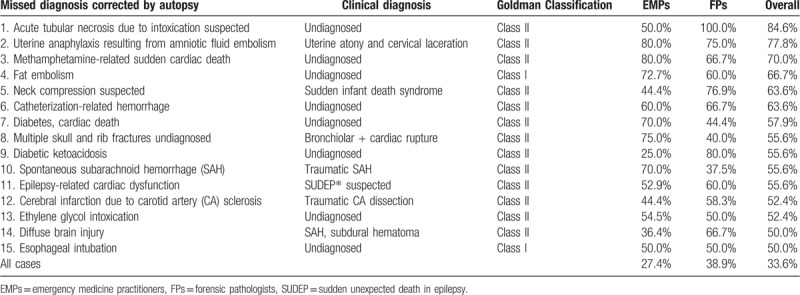
Correction rates of clinical-autopsy diagnoses by emergency medicine practitioners and forensic pathologists after the case conference.

High discrepancy rates were noted in cases of intoxication (3 cases: 84.6%, 70.0%, 50.0%), gynecological hemorrhage (77.8%), fat embolism (66.7%), asphyxia (63.6%), catheter-induced hemorrhage (63.6%), diabetes-associated death (2 cases: 57.9%, 55.6%), SAH (55.6%), chest-abdominal organ injuries (55.6%), brain infarction related to carotid artery sclerosis and atrial fibrillation (52.4%), and diffuse brain injury (50.0%). According to Goldman, major misdiagnoses should be categorized as class I (would have changed patient management, cure, or survival) or class II (would not have modified patient care).^[1]^ Major clinical misdiagnoses were corrected by autopsy in 15 cases: 2 class I cases (3.8%; fat embolism and esophageal intubation) and 13 class II cases (25%; Table 3).

SAH was the major imaging finding in 4 cases (Table 2). Case 1 presented with a headache but was discharged. An overlooked SAH was identified at the second admission. In this case, the discrepancy between the clinical and autopsy diagnoses was low (EMPs, 0%; FPs, 18%). However, all FPs but only 50% of EMPs noted the necessity of an autopsy, reflecting the difference in the recognition of a legal issue. Notably, all attendees admitted that autopsy was required except in case 1, despite the positive SAH findings. In case 2, imaging analysis revealed SAH and carotid artery sclerosis, both of which were identified as traumatic by autopsy. In case 3, imaging revealed SAH and a subdural hemorrhage but not a lethal diffuse brain injury (DBI). In case 3, most EMPs and FPs acknowledged the danger of a misleading diagnosis by imaging and the usefulness of autopsy, although the clinical-autopsy discrepancy rate was higher among FPs (66.7%) than among EMPs (36.4%). In case 4, although accident history supported the diagnosis of traumatic SAH, the autopsy findings corrected the diagnosis to spontaneous SAH in diagnoses provided by twice as many FMPs (70.0%) than in those provided by FPs (37.5%). Interestingly, the later the conference, the more EMPs were likely to correct clinical diagnoses in cases 2–4 involving violence or accidents.

## 4 Discussion

Previous reports have addressed the discrepancies between clinical and autopsy diagnoses as well as the necessity of autopsy for preventing misdiagnosis.^[1–3]^ Of the autopsy-corrected misdiagnoses in the EM and ICU cases in previous studies, the incidences of Goldman's classes I+II misdiagnoses were 31.7% and 19%, with autopsy rates of 53.0% and 97%, respectively.^[3,6]^ For the first time, we demonstrated the advantage of case discussions in conferences for forensic autopsy cases related to EM practices, in contrast with previous reports involving hospital autopsies based on natural deaths and MPADs. We found that the conference discussions on autopsy findings not only corrected diagnoses but also deepened the expertise and understanding of both the EMPs and FPs regarding the importance of autopsies. Additionally, the multi-disciplinary discussions of clinical and autopsy findings ensured accurate diagnoses, improvement of EM and FP practices, and prevention of similar accidents and false accusations. Major clinical misdiagnoses were corrected by considering autopsy findings in 33.6% of our cases, including 2 (3.8%) class I misdiagnoses (fat embolism and esophageal intubation). Retrospectively, all cases with a discrepancy rate of more than 50% between clinical and autopsy diagnoses (15 cases, 28.8%; Table 3) necessitated autopsy findings and conference discussions.

A shortage of FPs and low autopsy rates have promoted the idea that imaging can replace autopsies. Additionally, previous studies have shown the advantages of imaging with regard to the diagnoses of air embolus, pneumothorax, bullet/explosion injuries, remnant medical devices, and multiple fractures.^[6,7]^ However, a recent report on 182 serial forensic autopsy cases in the United Kingdom revealed that the highest discrepancy rates for causes of death as identified by imaging and autopsy were 32% for computed tomography (CT) and 43% for magnetic resonance imaging.^[8]^ In our 4 cases with SAH as the major imaging finding (Table 2), the discrepancy rates between clinical and autopsy diagnoses made by EMPs increased in the later conferences, suggesting the learning effects of the conferences. Consistent with our interpretation, a study on the effects of communication between clinicians and pathologists in 256 autopsy cases over 5 years demonstrated that the discrepancy rate in the death cause between clinical and autopsy diagnoses decreased from 43% to 26% overall and from 30% to 17% in class I misdiagnoses.^[9]^ In case 3, imaging analysis revealed an SAH and subdural hemorrhage but not a lethal DBI, indicating the risk of false-positive and false-negative diagnoses that may result from relying on imaging techniques. In case 4, which involved a traffic accident, the diagnosis was corrected from traumatic SAH to spontaneous SAH by twice as many EMPs as by FPs after the discussion of autopsy findings (70.0% vs. 37.5%). Previously, we conducted a questionnaire study to determine whether intracranial injury diagnoses should be excluded if no brain CT abnormalities had been identified. EMPs were much more likely to provide positive answers (55%) than FPs (9%).^[4]^ However, except for the EMPs who responded to case 1 (spontaneous SAH), all attendees acknowledged the necessity of autopsy despite the positive image findings for SAH (particularly in cases of accidents or violence). These cases prompted the EMPs to notice that imaging can mislead their diagnoses. The EMPs may have also learned that loss of consciousness related to natural diseases, including heart attack, epilepsy, or diabetes, can lead to traumatic SAH and that injuries resulting from falls or accidents may trigger spontaneous SAH.

An autopsy is warranted in unwitnessed cases, accident cases, and cases with potential involvement of third-party individuals or future legal issues, even if imaging indicates SAH or other lesions, as implied by our study results. Moreover, such cases remind EMPs of the importance of autopsies in discriminating traumatic and spontaneous SAH, detecting non-visible injuries, and excluding external causes of death apart from positive imaging findings. This article is the first to highlight the concrete implications of forensic autopsy in the diagnosis of SAH, which is usually handled without autopsy by many clinicians.

The discrepancy between clinical and autopsy diagnoses is a critical issue in medico-legal cases, as misdiagnoses can lead to false accusations on crime suspects and on doctors suspected of medical mishaps. In one case in this study, an initial clinical diagnosis of traumatic carotid artery (CA) dissection resulting in brain infarction misled lawyers to falsely accuse a suspect of violence. Shortly thereafter, the patient died, and a medico-legal autopsy was conducted. The autopsy findings, histology, and conference discussion excluded a traumatic CA dissection and supported the diagnosis of a pre-existing thrombus with severe CA atheroma and atrial fibrillation as the cause of brain infarction. As a result, the suspect was spared from false accusation on a charge of injury resulting in death. In another case of fatal cardiac tamponade during an ablation procedure, the initial expert opinion of catheter-induced perforation was corrected to rupture following myocardial infarction at the conference. Correcting the diagnosis prevented false prosecution of the involved physician for malpractice. These cases deeply impressed prosecutors who attended the conferences and responded by stating that they would have falsely prosecuted the suspects if they had not attended the conference. In Japan, a number of doctors have been falsely prosecuted on the basis of opinions of medical experts with insufficient experience in the concerned field. The conference approach could prevent such false accusations on medical practitioners through education of both medical and legal experts.

With the permission of the prosecutors, we requested that police officers provide the autopsy information and issues addressed during the conference to practitioners related to each case. In such cases, the doctors often responded quickly to provide explanations for the questionable practices. Notably, several doctors stated that they improved their prevention procedures. However, the most serious drawback of medico-legal autopsy in Japan is that by law, the information cannot be disclosed to the concerned medical practitioners or explained adequately to the bereaved families.^[5,10–13]^ The Criminal Procedure Code prohibits the disclosure of information related to medico-legal autopsy before disclosure in a criminal court or before a prosecutor decides to prosecute. However, regarding serious cases of MPADs, the prosecutor can demand that FPs not respond to civil court requests to disclose information about their medico-legal autopsies. Previously, we found that most bereaved families whose next-of-kin underwent medico-legal autopsies sued the involved doctors because autopsy information had not been disclosed to them.^[5,10–12]^ Legal reformation regarding disclosure of autopsy information is difficult in Japan. However, the legal authority should be notified about the necessity of disclosure of clinical and autopsy information to third-party EMPs and FPs in case conferences for accurate and fair diagnoses, as well as for public interest. The conference approach would benefit not only EMPs and FPs but also attending practitioners, bereaved families, and prosecutors. The law nominally acknowledges disclosure for public interest. More efforts are required to promote the public interest merits of the autopsy and conference approach in terms of its ability to provide fair and scientific expert opinions as well as to prevent similar accidents and false prosecution. In support of this idea, a questionnaire study showed that prior exposure to autopsy was shown to strengthen a clinician's belief in the relevance of autopsy in improving diagnostic accuracy and quality of medical practices.^[14]^ The clinician's belief that autopsy will increase autopsy rates and foster the recognition of importance of autopsy information and conferences is important not only for clinicians but also for lawyers and for the society in general.

## 5 Conclusions

Autopsy case-based conferences improved the accuracy and expertise of diagnoses provided by EM and FP practitioners. The usefulness of imaging for autopsy was acknowledged by two thirds of the attendees. However, our results also suggested that imaging cannot replace autopsy in deaths related to procedure or violence and in several categories of deaths such as those related to intoxication, asphyxia, diabetes, and subarachnoid hemorrhage.

## Acknowledgments

We appreciate the cooperation of the Japanese Association for Acute Medicine. We also thank Editage for English language editing.

## Author contributions

**Conceptualization:** Hideyuki Maeda, Takako Tsujimura.

**Data curation:** Hideyuki Maeda, Takako Tsujimura.

**Formal analysis:** Hideyuki Maeda.

**Investigation:** Hideyuki Maeda.

**Methodology:** Hideyuki Maeda, Takako Tsujimura, Ken-ichi Yoshida.

**Project administration:** Hideyuki Maeda.

**Resources:** Hideyuki Maeda.

**Software:** Hideyuki Maeda.

**Supervision:** Ken-ichi Yoshida.

**Validation:** Hideyuki Maeda.

**Visualization:** Hideyuki Maeda, Ken-ichi Yoshida.

**Writing – original draft:** Hideyuki Maeda, Ken-ichi Yoshida.

**Writing – review & editing:** Hideyuki Maeda, Ken-ichi Yoshida.
